# UNC5A IgG in a Previously Reported CASPR2/LGI1‐Negative VGKC‐Associated Peripheral Nerve Hyperexcitability Syndrome: Long‐Term Clinical and Electrophysiological Follow‐Up

**DOI:** 10.1155/crnm/5816975

**Published:** 2026-10-04

**Authors:** Birte Eikeland

**Affiliations:** ^1^ Primary Health Clinic Bergen Municipality Bergen Norway

## Abstract

Autoimmune peripheral nerve hyperexcitability (PNH) syndromes are most commonly associated with antibodies against CASPR2 or LGI1. However, a subset of patients with clinically and electrophysiologically confirmed PNH remains negative for these established neuronal surface antibodies despite persistent VGKC antibody positivity. This report presents the long‐term follow‐up of a patient previously diagnosed with a Morvan‐like autoimmune PNH syndrome following enteroviral infection. Serial neurophysiological studies demonstrated persistent motor axonal hyperexcitability, while treatment with intravenous immunoglobulin (IVIg) resulted in sustained clinical stabilization and substantial functional improvement. Additional research‐based immunological analyses identified UNC5A IgG reactivity in serum using a live cell‐based assay. UNC5A antibodies have previously been identified predominantly in the context of thymoma‐associated neuromyotonia, including patients with Morvan syndrome and myasthenia gravis, often in association with CASPR2 antibodies. The identification of UNC5A IgG in a patient with electrophysiologically confirmed CASPR2/LGI1‐negative PNH and persistent VGKC antibodies represents a hypothesis‐generating association rather than evidence of a causal or pathogenic role. UNC5A IgG may represent an associated biomarker or a secondary immune phenomenon within a broader autoimmune response. This observation extends the clinical context in which UNC5A reactivity has been identified and supports further investigation of UNC5A and other candidate neuronal surface antigens in CASPR2/LGI1‐negative PNH.

## 1. Introduction

Peripheral nerve hyperexcitability (PNH) syndromes are characterized by spontaneous motor axon discharges resulting in fasciculations, myokymia, and neuromyotonia and are associated with dysfunction of voltage‐gated potassium channels (VGKC) [1]. Autoimmune forms are commonly linked to antibodies targeting proteins within the VGKC complex, particularly CASPR2 and LGI1 [2, 3]. However, autoimmune PNH may occur despite negative testing for CASPR2 and LGI1 antibodies [2, 3].

In some of these patients, persistent low‐titer VGKC antibodies detected by radioimmunoassay (RIA) may still be present, although the clinical significance of VGKC seropositivity in the absence of CASPR2 or LGI1 specificity remains debated [4, 5]. Previous studies of Morvan syndrome and recent reviews have suggested that additional, as yet unidentified, neuronal surface antibodies may be present in a subset of CASPR2/LGI1‐negative PNH syndromes [2, 3, 6].

The patient was previously reported to have a Morvan‐like autoimmune PNH syndrome characterized by motor manifestations of PNH, dysautonomia, sensory small‐fiber involvement with neuropathic pain, and central nervous system manifestations following enteroviral infection [7]. The patient demonstrated persistent low‐titer VGKC antibodies by RIA despite negative testing for CASPR2 and LGI1.

The present report extends the previously reported case by providing long‐term clinical follow‐up, serial electrophysiological documentation of motor axonal hyperexcitability, and new research‐based immunological findings, including the identification of UNC5A IgG by live cell‐based assay. Although the pathogenic significance of UNC5A IgG remains uncertain, its identification represents a hypothesis‐generating immunological association and raises the possibility that additional neuronal surface antibodies may be present in selected CASPR2/LGI1‐negative PNH syndromes.

## 2. Case Presentation

The initial clinical presentation and diagnostic evaluation have been described in detail previously [7]. Briefly, the patient was a 42‐year‐old woman who developed a postinfectious neurological syndrome approximately one month after enteroviral pericarditis in 2006. Initial manifestations included severe neuropathic pain and allodynia, painful muscle cramps, fasciculations, proximal muscle weakness, and marked functional impairment, resulting in a period of bed‐bound status.

The condition was initially considered a postinfectious immunological reaction with muscular involvement that was expected to be self‐limiting, although recovery was considered potentially prolonged and could take up to 1 year. Because of the severity of the symptoms, prednisone 40 mg/day was initiated and tapered over approximately 6 months, without significant clinical benefit.

Gradual spontaneous improvement subsequently occurred, but functional capacity remained markedly reduced, initially because of neuropathic pain and painful muscle cramps, and later also because of increasing muscle weakness and stiffness. Neuropathic pain has previously been described in VGKC‐complex autoimmunity, including in patients without identified CASPR2 or LGI1 antibodies, and may reflect involvement of small sensory axons [8]. Over the following years, a few distinct exacerbations occurred, typically following excessive physical activity, with substantial temporary loss of previously regained function.

In 2010, however, the clinical phenotype changed substantially, with prominent generalized muscle stiffness and dystonic posturing, myoclonic‐like movements, fasciculations, painful paresthesias, and muscle cramps, accompanied by CNS manifestations of severe insomnia and agitation and autonomic symptoms including facial flushing and excessive sweating.

During a 4‐week admission to a psychiatric department, the presentation was interpreted as a somatoform disorder. The patient was subsequently admitted to a neurological department, where the somatoform diagnosis was maintained. Nerve conduction studies demonstrated tibial F‐wave findings that were interpreted as normal at the time but later recognized as suggestive of PNH (Supporting Figure 1), while EMG showed only nonspecific abnormalities. ANA and RNP antibodies, which had been negative in 2007, were found to be positive during this evaluation. In the context of the prevailing somatoform diagnosis, PNH was not recognized, VGKC antibodies by RIA were not tested, and further neurological investigation was limited, delaying recognition of the neurological disorder and consideration of immunotherapy.

Despite the severity of the 2010 exacerbation, slow spontaneous improvement occurred over the following four years without major relapses, with walking capacity increasing from approximately 50 m in 2011 to around 2–3 km by 2014. In 2014, substantially increased walking activity was followed by an acute and severe relapse, with marked worsening of neuropathic pain, fasciculations, painful muscle cramps, and muscle stiffness, resulting in profound loss of mobility. The patient was largely immobilized for 7–8 months and required a wheelchair during this period. Exercise‐induced worsening has previously been described in PNH syndromes [9].

VGKC antibodies were first identified by RIA in May 2015 at 100 pmol/L (reference < 72 pmol/L) at EUROIMMUN, Lübeck, Germany, while LGI1 and CASPR2 antibodies were negative.

In the context of the severe 2014–2015 relapse, the recurrent clinical phenotype, and the presence of VGKC, ANA, and RNP antibodies, an autoimmune neurological etiology was reconsidered. Although PNH had not yet been electrophysiologically confirmed, the combined clinical and immunological findings provided the rationale for a therapeutic trial of IVIg, which was initiated in December 2015 at 2 g/kg (150 g over 5 days) and repeated at the same total dose on two further occasions in 2016.

Four weeks after the third high‐dose IVIg course, administered in June 2016, the VGKC antibody level had decreased from the pretreatment level of 100 pmol/L to 10 pmol/L, within the reference range. At the time, this marked reduction was considered by the evaluating neurologist to be consistent with a treatment effect of IVIg. Subsequent measurements remained variably positive, generally ranging from approximately 60–100 pmol/L. The most recent level, measured on February 4, 2025, four weeks after the preceding 40‐g IVIg infusion, was 88 pmol/L. ANA and RNP antibodies remained positive on subsequent testing; at the most recent assessment in January 2026, ANA was positive and RNP was > 8.

Despite persistent ANA and RNP positivity, marked Raynaud’s phenomenon, and mild inflammatory changes on muscle biopsy, rheumatological evaluation did not identify a systemic rheumatic disease. The patient had also been evaluated for possible Sjögren’s syndrome in young adulthood, but the diagnosis was not confirmed.

In 2016, the patient underwent further evaluation at a highly specialized referral center in the United States. Thermoregulatory sweat testing demonstrated patchy hypohidrosis affecting the calves, thighs, and both arms, with findings suggestive of small‐fiber neuropathy (Supporting Figure 2). Based on the overall clinical presentation and immunological findings, including VGKC, ANA, and RNP antibodies, the condition was assessed as “*immune responsive VGKC-associated autoimmunity, with multiple immune inflammatory markers*,” and continued IVIg treatment was recommended.

The diagnosis of PNH was subsequently electrophysiologically confirmed by EMG in 2019, with findings consistent with PNH (Figure 1). Further EMG examination in 2022 demonstrated persistent pathological findings of PNH (Figure 2), with subsequent examinations in 2025–2026 continuing to demonstrate the electrophysiological abnormality.

**FIGURE 1:**
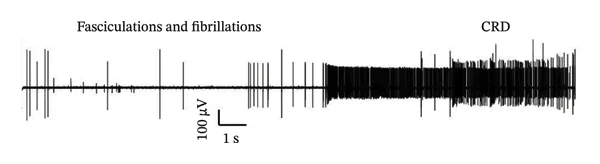
Needle electromyography demonstrating motor axonal hyperexcitability in 2019. Representative recording from the right abductor pollicis brevis muscle showing fasciculations, fibrillation potentials, and complex repetitive discharges (CRDs) at 90–120 Hz. Published with permission from the department of clinical neurophysiology, Rigshospitalet, Copenhagen, Denmark.

**FIGURE 2:**
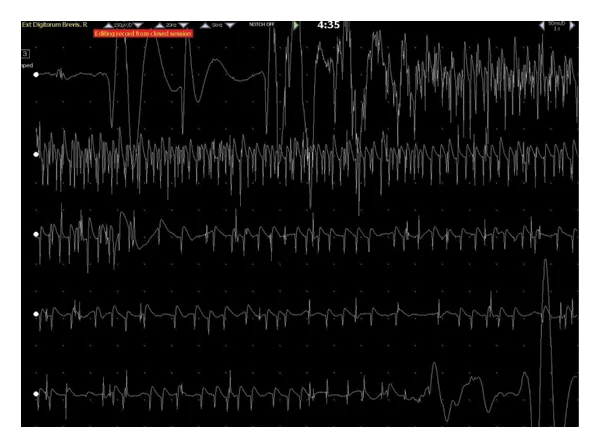
Follow‐up needle electromyography demonstrating persistent motor axonal hyperexcitability in 2022. Representative recording from the right extensor digitorum brevis muscle showing spontaneous pseudo‐myotonic discharges consistent with ongoing motor axonal hyperexcitability. Published with permission from the department of clinical neurophysiology, Haukeland University Hospital, Bergen, Norway.

In August 2016, maintenance IVIg was initiated at 30 g every 4 weeks and subsequently increased, generally to 60–90 g every 4 weeks, because of insufficient control of stiffness and gait impairment. With sustained clinical improvement, IVIg was gradually tapered from 2022 by extending treatment intervals and subsequently reducing the dose. Treatment was discontinued in September 2025, with no clinical relapse to date. No concomitant immunotherapy was used during long‐term IVIg treatment. This individualized, response‐guided treatment approach is consistent with previous recommendations that IVIg frequency and duration in PNH may be tailored according to clinical response [10].

During the first few years of the disease, symptomatic treatment included intermittent use of paracetamol/codeine and oxazepam, but neither was part of the subsequent long‐term treatment. During the psychiatric admission in 2010, quetiapine was initiated at doses up to approximately 400 mg/day. After the acute phase, the dose was substantially reduced and has since been continued at 25 mg nightly for sleep.

Clinical improvement was most evident in muscle stiffness, neuropathic pain, posture, gait, and overall physical function, as well as in autonomic symptoms, particularly facial flushing. Physiotherapy assessments before IVIg initiation and after the three high‐dose courses documented substantial improvement in stiffness, posture, gait, and physical function. During maintenance treatment, pain and muscle stiffness persisted but tended to worsen with longer treatment intervals. Over time, functional capacity improved substantially, allowing a return to independent daily activities and, from 2016, professional work at approximately 60% of full‐time capacity.

At the most recent follow‐up, the patient remained clinically stable off immunotherapy, with mild residual stiffness, fasciculations/myokymia, and neuropathic sensory symptoms. Functional independence was preserved, with a walking capacity of approximately 3–4 km and the ability to swim approximately 400 m three to four times per week.

Longitudinal neurophysiological and autonomic investigations provided objective evidence of PNH and autonomic involvement at different stages of the disease. In 2010, nerve conduction studies demonstrated tibial F‐wave findings that were interpreted as normal at the time but later recognized as suggestive of PNH (Supporting Figure 1). Concurrent needle EMG showed only limited nonspecific abnormalities without recognized motor hyperexcitability. Thermoregulatory sweat testing in 2016 demonstrated patchy hypohidrosis affecting the calves, thighs, and both arms, providing objective evidence of autonomic small‐fiber involvement (Supporting Figure 2).

Motor axonal hyperexcitability was subsequently demonstrated by needle EMG in 2019, with fasciculations, fibrillation potentials, and complex repetitive discharges (CRDs), as illustrated in Figure 1. Follow‐up EMG in 2022 demonstrated increased insertional activity, myokymic and pseudo‐myotonic discharges, and CRDs; Figure 2 shows a representative recording of the pseudo‐myotonic discharges. Subsequent examinations in 2025–2026 continued to show motor hyperexcitability.

Additional research‐based immunological investigations identified UNC5A IgG reactivity in serum using a live cell‐based assay at a dilution of 1:40; CSF was not tested. The serum sample was obtained four weeks after the preceding 40‐g IVIg infusion. Reactivity was assessed semiquantitatively on a 0–4 staining scale (0, no staining; 1, faint staining; 2, fair staining; 3, strong staining; 4, very strong staining). The initial analysis yielded a score of 2/4, and repeat testing of the same serum sample yielded a score of 1.5/4. Antibody titration was not performed.

Extensive testing for antibodies against other candidate antigens was negative, including CASPR2, LGI1, contactin‐2, potassium channel subunits (Kv1, Kv2, and Kv6), ADAM23, and LGI3. No thymoma was identified.

Additional exploratory research‐based immunofluorescence studies performed independently in two laboratories using patient serum on experimental peripheral nerve tissue demonstrated IgM reactivity associated with peripheral nerve structures. In one laboratory, patient serum was applied to mouse sciatic nerve tissue, demonstrating IgM reactivity associated with Schwann cell‐associated structures surrounding unmyelinated C‐fibers (Supporting Figure 3). The IgM binding was described by the investigating laboratory as moderate; quantitative analysis was not performed. In the other laboratory, patient serum was examined on monkey peripheral nerve tissue, demonstrating IgM reactivity associated with myelin in both small and large myelinated nerve fibers.

Further antigen identification was not pursued because the staining lacked a sufficiently distinctive localization to suggest a specific candidate antigen, and available immunoprecipitation‐based approaches were considered poorly suited to identifying antigens targeted by IgM. These exploratory findings were not quantitatively validated, and their antigenic specificity and pathogenic significance therefore remain uncertain.

## 3. Discussion

UNC5A antibodies have previously been identified predominantly in the context of thymoma‐associated neuromyotonia, including patients with Morvan syndrome and myasthenia gravis, often in association with CASPR2 antibodies [11, 12]. More recently, systematic live CBA testing for UNC5A alongside CASPR2, LGI1, and contactin‐2 in an NMT cohort in which most patients had thymoma and/or MG identified UNC5A IgG in three patients, including one with isolated UNC5A reactivity [13]. In contrast, UNC5A IgG was identified here in a patient without thymoma, with electrophysiologically confirmed PNH, persistent low‐titer VGKC antibodies, negative CASPR2 and LGI1 antibodies, and extensive negative testing for other candidate VGKC‐associated antigens.

Importantly, UNC5A reactivity was identified using a live cell‐based assay that detects antibodies binding to extracellularly accessible antigens on living cells, a methodological approach used to identify neuronal surface autoantibodies [14].

This case highlights the limitations of classifying autoimmune PNH syndromes solely on the basis of currently recognized neuronal surface antibodies. PNH syndromes comprise a heterogeneous group of disorders in which autoimmune mechanisms are increasingly recognized. However, some patients with autoimmune PNH remain negative for CASPR2 and LGI1 antibodies [2, 3].

Although VGKC antibodies lacking CASPR2 or LGI1 specificity are often regarded as clinically nonspecific [4, 5], the present patient demonstrated a clinical phenotype consistent with autoimmune PNH, with objective electrophysiological evidence of PNH, a relapsing clinical course, and sustained clinical stabilization during IVIg treatment. These findings illustrate the importance of interpreting VGKC antibody positivity within the clinical and electrophysiological context.

This observation is consistent with previous studies of Morvan syndrome demonstrating that some clinically typical patients lack CASPR2 and LGI1 antibodies but show additional neuronal surface immunoreactivity suggestive of unidentified antigenic targets [6]. Recent reviews have similarly emphasized that patients with autoimmune PNH may test negative for CASPR2 and LGI1, raising the possibility of additional neuronal surface autoantibodies [2, 3]. In addition, it has been suggested that VGKC antibodies lacking CASPR2 or LGI1 specificity may occasionally coexist with additional cell‐surface autoantibodies, particularly in neuromyotonia, providing a rationale for continued investigation of novel extracellular antigenic targets [4].

In this context, the role of Netrin–UNC5 signaling in Schwann cell–axon interactions in the peripheral nervous system provides a potential biological context for the UNC5A finding [15]. Kv1 potassium channels are concentrated at juxtaparanodal regions of myelinated axons, where their localization and stabilization depend on membrane‐organizing proteins including CASPR2, contactin‐2, and ADAM23 [16–18]. Disruption of these structures may impair Kv1 channel stabilization and increase susceptibility to spontaneous axonal activity [17]. However, the UNC5A finding in this single case should be regarded as a hypothesis‐generating association rather than evidence of a causal or pathogenic role. UNC5A IgG may represent an associated biomarker or a secondary immune phenomenon within a broader autoimmune response. Nevertheless, given the biological role of Netrin–UNC5 signaling and the importance of membrane organization for Kv1 channel function, a potential relationship between UNC5A IgG reactivity and neuronal hyperexcitability warrants further investigation [15–18].

Thermoregulatory sweat testing demonstrated patchy hypohidrosis affecting the calves, thighs, and both arms (Supporting Figure 2), providing objective evidence of autonomic small‐fiber involvement in addition to the persistent motor axonal hyperexcitability. The involvement of multiple components of the nervous system, including motor, sensory, autonomic, and central nervous system manifestations, is consistent with the recognized clinical spectrum of autoimmune PNH [6, 9].

Additional immunofluorescence studies performed independently in two research laboratories demonstrated IgM reactivity associated with peripheral nerve structures. In the absence of clinical or electrophysiological evidence of demyelination, these findings do not support a primary IgM‐mediated demyelinating neuropathy. Together with the presence of other autoimmune markers, they may reflect broader immune activation; however, given their uncertain antigenic specificity and pathogenic significance, they should not be interpreted as evidence of a disease‐specific pathogenic mechanism.

The Morvan‐like phenotype, persistent low‐titer VGKC antibody positivity, objective long‐term electrophysiological evidence of motor axonal hyperexcitability, and sustained clinical response to IVIg support the diagnosis of an autoimmune PNH syndrome despite persistent CASPR2/LGI1 negativity. The subsequent identification of UNC5A IgG represents an additional immunological observation of uncertain significance in this context.

Taken together, this case supports further investigation of UNC5A and other candidate neuronal surface antigens in patients with autoimmune PNH who remain negative for established neuronal surface antibodies.

## Funding

No funding was received for this manuscript.

## Consent

Written informed consent for publication of this case report and any accompanying images was obtained from the patient.

## Conflicts of Interest

The author declares no conflicts of interest.

## Supporting Information

Additional supporting information can be found online in the Supporting Information section.

## Supporting information


**Supporting Information** Supporting Figure 1. F‐wave studies demonstrating early motor axonal hyperexcitability on tibial nerve conduction studies performed during the period of greatest clinical severity in 2010. Supporting Figure 2. Thermoregulatory sweat testing demonstrating patchy hypohidrosis, providing objective evidence of autonomic small‐fiber dysfunction. Supporting Figure 3. Immunofluorescence analyses performed independently in two research laboratories demonstrating IgM reactivity associated with peripheral nerve structures, including myelin‐associated structures and Schwann cell‐associated structures compatible with unmyelinated C‐fibers.

## Data Availability

The data that support the findings of this study are available on request from the corresponding author. The data are not publicly available due to privacy or ethical restrictions.
