# Supplementary material for: UNC5A IgG in a Previously Reported CASPR2/LGI1‐Negative VGKC‐Associated Peripheral Nerve Hyperexcitability Syndrome: Long‐Term Clinical and Electrophysiological Follow‐Up
Source: Case Rep Neurol Med. 2026 Oct 4;2026:5816975. doi: 10.1155/crnm/5816975 (PMC13635785; doi:10.1155/crnm/5816975)
Supplement: Supplementary file 1 [file CRNM-2026-5816975-s001.docx]

**Supplementary Materials**

**Supplementary Figure 1**

F-wave studies demonstrating abnormalities suggestive of peripheral nerve hyperexcitability on nerve conduction studies in 2010.

**Supplementary Figure 2**

Thermoregulatory sweat testing in 2016 demonstrated patchy hypohidrosis affecting the calves, thighs, and both arms, with findings suggestive of small-fiber neuropathy.

**Supplementary Figure 3**
Immunofluorescence analysis of teased mouse sciatic nerve fibers demonstrating IgM immunoreactivity associated with Schwann cell-associated structures surrounding unmyelinated C-fibers.

**Upper panel:** IgM immunoreactivity (white) associated with Schwann cell-associated structures surrounding unmyelinated C-fibers.

**Lower panel:** Triple immunofluorescence labeling showing IgM (red), voltage-gated sodium channel clusters (Nav, green), and neurofilament heavy chain (NF-H, blue). IgM immunoreactivity was associated with Schwann cell-associated structures surrounding unmyelinated C-fibers without clear co-localization with axonal markers. The asterisk indicates a representative unmyelinated nerve fiber demonstrating Schwann cell-associated IgM immunoreactivity.

*Images courtesy of Jérôme Devaux, used with permission.*
